# Supplementary material for: The automated Galaxy-SynBioCAD pipeline for synthetic biology design and engineering
Source: Nat Commun. 2022 Aug 29;13:5082. doi: 10.1038/s41467-022-32661-x (PMC9424320; doi:10.1038/s41467-022-32661-x)
Supplement: Supplementary file 2 — Description of Additional Supplementary Files [file 41467_2022_32661_MOESM2_ESM.pdf]

Supplementary Dataset 1: Workflow and experimental data for the Lycopene benchmarking.

Supplementary Dataset 2: Literature pathways and associated matching scores.

Supplementary Dataset 3: Training sets and predicted scores used for pathway rankings.
