# Supplementary material for: The automated Galaxy-SynBioCAD pipeline for synthetic biology design and engineering
Source: Nat Commun. 2022 Aug 29;13:5082. doi: 10.1038/s41467-022-32661-x (PMC9424320; doi:10.1038/s41467-022-32661-x)
Supplement: Supplementary file 3 — Reporting Summary [file 41467_2022_32661_MOESM3_ESM.pdf]

nature portfolio

Corresponding author(s): Jean-Loup Faulon

Last updated by author(s): 29/07/2022

Reporting Summary

Nature Portfolio wishes to improve the reproducibility of the work that we publish. This form provides structure for consistency and transparency in reporting. For further information on Nature Portfolio policies, see our Editorial Policies and the Editorial Policy Checklist.

Statistics

For all statistical analyses, confirm that the following items are present in the figure legend, table legend, main text, or Methods section.

n/a	Confirmed	
☒	☐	The exact sample size (*n*) for each experimental group/condition, given as a discrete number and unit of measurement
☒	☐	A statement on whether measurements were taken from distinct samples or whether the same sample was measured repeatedly
☒	☐	The statistical test(s) used AND whether they are one- or two-sided<br>*Only common tests should be described solely by name; describe more complex techniques in the Methods section.*
☒	☐	A description of all covariates tested
☒	☐	A description of any assumptions or corrections, such as tests of normality and adjustment for multiple comparisons
☒	☐	A full description of the statistical parameters including central tendency (e.g. means) or other basic estimates (e.g. regression coefficient) AND variation (e.g. standard deviation) or associated estimates of uncertainty (e.g. confidence intervals)
☒	☐	For null hypothesis testing, the test statistic (e.g. *F*, *t*, *r*) with confidence intervals, effect sizes, degrees of freedom and *P* value noted<br>*Give P values as exact values whenever suitable.*
☒	☐	For Bayesian analysis, information on the choice of priors and Markov chain Monte Carlo settings
☒	☐	For hierarchical and complex designs, identification of the appropriate level for tests and full reporting of outcomes
☒	☐	Estimates of effect sizes (e.g. Cohen's *d*, Pearson's *r*), indicating how they were calculated

Our web collection on statistics for biologists contains articles on many of the points above.

Software and code

Policy information about availability of computer code

Data collection	No software was used for data collection.
Data analysis	The following data analysis tools are open source codes on GitHub and on the Galaxy ToolShed (links are provided):<br>- rrparser (source code: https://github.com/brsynth/RRParser, Galaxy Toolshed node: https://toolshed.g2.bx.psu.edu/view/tduigou/rrparser/ea590c609fec , version: 2.4.6)<br>- rpextractsink (source code: https://github.com/brsynth/rptools/tree/master/rptools/rpextractsink, Galaxy Toolshed node: https://toolshed.g2.bx.psu.edu/view/tduigou/rpextractsink/47bb93e7832b , version: 5.12.1)<br>- retropath2 (source code: https://github.com/brsynth/RetroPath2-wrapper, Galaxy Toolshed node: https://toolshed.g2.bx.psu.edu/view/tduigou/retropath2/9c8ac9980bd6 , version: 2.3.0)<br>- rp2paths (source code: https://github.com/brsynth/rp2paths, Galaxy Toolshed node: https://toolshed.g2.bx.psu.edu/view/tduigou/rp2paths/e3db7fb2c85e, version: 1.5.0)<br>- rpcompletion (source code: https://github.com/brsynth/rptools/tree/master/rptools/rpcompletion, Galaxy Toolshed node: https://toolshed.g2.bx.psu.edu/view/tduigou/rpcompletion/b8242cf18cc0 , version: 5.12.1)<br>- rpthermo (source code: https://github.com/brsynth/rptools/tree/master/rptools/rpthermo , Galaxy Toolshed node: https://toolshed.g2.bx.psu.edu/view/tduigou/rpthermo/21a900eee812 , version: 5.12.1)<br>- rpfba (source code: https://github.com/brsynth/rptools/tree/master/rptools/rpfba , Galaxy Toolshed node: https://toolshed.g2.bx.psu.edu/view/iuc/rpfba/19be43e96154, version: 5.12.1)<br>- rpscore (source code: https://github.com/brsynth/rptools/tree/master/rptools/rpscore , Galaxy Toolshed node: https://toolshed.g2.bx.psu.edu/view/tduigou/rpscore/da8ae7fa5ed3 , version: 5.12.1)<br>- rpranker (source code: https://github.com/brsynth/rptools/tree/master/rptools/rprank , Galaxy Toolshed node: https://toolshed.g2.bx.psu.edu/view/tduigou/rpranker/e95370d2e5f9 , version: 5.12.1)<br>- rpreport (source code: https://github.com/brsynth/rptools/tree/master/rptools/rpreport , Galaxy Toolshed node: https://toolshed.g2.bx.psu.edu/view/tduigou/rpreport/d09a51507aaf , version: 5.12.1)

- rpviz (source code: https://github.com/brsynth/rptools/tree/master/rptools/rpviz , Galaxy Toolshed node: https://toolshed.g2.bx.psu.edu/view/tduigou/rpviz/ea2ca40a24c5 , version: 5.10.0)
- selenzy (source code: https://github.com/pablocarb/selenzy , Galaxy Toolshed node: https://toolshed.g2.bx.psu.edu/view/tduigou/selenzy/34a9d136a5bf , version: 0.2.0)
- sbml2sbol (source code: https://github.com/neilswainston/SbmlToSbol , Galaxy Toolshed node: https://toolshed.g2.bx.psu.edu/view/tduigou/sbml2sbol/83108f3c65aa , version: 0.1.13)
- partsgenie (source code: https://github.com/neilswainston/PartsGenieClient , Galaxy Toolshed node: https://toolshed.g2.bx.psu.edu/view/tduigou/partsgenie/295a21fc55d0 , version: 1.0.1)
- optdoe (source code: https://github.com/pablocarb/doebase , Galaxy Toolshed node: https://toolshed.g2.bx.psu.edu/view/tduigou/optdoe/c3f32929a4b7 , version: 2.0.2)
- dnaweaver (source code: https://github.com/brsynth/DNAWeaver_SynBioCAD , Galaxy Toolshed node: https://toolshed.g2.bx.psu.edu/view/tduigou/dnaweaver/c519517e3ade , version: 1.0.2)
- lcrgenie (source code: https://github.com/neilswainston/LCRGenie , Galaxy Toolshed node: https://toolshed.g2.bx.psu.edu/view/tduigou/lcrgenie/afbbecdcc0e3 , version: 1.0.2)
- rpbasicdesign (source code: https://github.com/brsynth/rpbasicdesign , Galaxy Toolshed node: https://toolshed.g2.bx.psu.edu/view/tduigou/rpbasicdesign/de9f53630349 , version: 0.3.4)
- dnabot (source code: https://github.com/BASIC-DNA-ASSEMBLY/DNA-BOT, Galaxy Toolshed node: https://toolshed.g2.bx.psu.edu/view/tduigou/dnabot/de6812daba39, version: 3.1.0)
- get_sbml_model (Galaxy Toolshed node: https://toolshed.g2.bx.psu.edu/view/tduigou/get_sbml_model/4797d0b36ff3, version: 0.0.1)

For manuscripts utilizing custom algorithms or software that are central to the research but not yet described in published literature, software must be made available to editors and reviewers. We strongly encourage code deposition in a community repository (e.g. GitHub). See the Nature Portfolio guidelines for submitting code & software for further information.

Data

Policy information about availability of data

All manuscripts must include a data availability statement. This statement should provide the following information, where applicable:
- Accession codes, unique identifiers, or web links for publicly available datasets
- A description of any restrictions on data availability
- For clinical datasets or third party data, please ensure that the statement adheres to our policy

Source data of figures are provided in the Source Data file. The eQuilibrator database is available online at Zenodo (https://doi.org/10.5281/zenodo.4128543) and it is queryable using the equilibrator-api python library which is available online at github (https://gitlab.com/equilibrator/equilibrator-api). The LASER database is available online at bitbucket (https://bitbucket.org/jdwinkler/laser_release/src/master/). All other relevant data are included in the paper and in the Supplementary Text and Dataset files.

Field-specific reporting

Please select the one below that is the best fit for your research. If you are not sure, read the appropriate sections before making your selection.

☒ Life sciences ☐ Behavioural & social sciences ☐ Ecological, evolutionary & environmental sciences

For a reference copy of the document with all sections, see nature.com/documents/nr-reporting-summary-flat.pdf

Life sciences study design

All studies must disclose on these points even when the disclosure is negative.

Sample size	Expert validation trial benchmarking: 40 samples of 5 pathways (200 total) were drawn randomly among 7919 generated pathway by Galaxy-SynBioCAD. No statistical analysis was performed on the samples, the samples were used to label pathways for the training set. To help in the visual inspection of the sets of pathways and keep achievable expert validation, the sample size sent to each expert was limited to 5.
Data exclusions	Expert validation trial benchmarking: no data was excluded.
Replication	Benchmarking workflows for lycopene production: all pathways engineered for lycopene production were replicated at two different locations (Paris and London). Experiments between the two locations have been performed independently. The spotting step was performed twice with different volumes in London, as reported in the paper. In the scope of this study, no other replication not reported in the paper has been performed.
Randomization	Expert validation trial benchmarking: cf. sample size comments, 40 samples of 5 pathways (200 total) were drawn randomly among 7919 generated pathway by Galaxy-SynBioCAD. Within a sample, the predicted pathways best matching the literature pathways (when known) was included and the four remaining pathways were drawn randomly. Samples were randomly assigned to experts.<br><br>Lycopene production benchmarking: each construct represents an independent individual, built in its specific plate well. There is no sample allocation into experimental groups.
Blinding	Expert validation trial benchmarking: we used a double-blinded strategy, neither the participants nor the conductors were aware of the origin of the pathways, and the trial participants were asked to flag pathways they deemed valid without having explicit information on pathways found in the literature.

Lycopene production benchmarking: instructions for building the constructs provided by python executable scripts were performed by liquid handling robots independently of the experimentalists.

Reporting for specific materials, systems and methods

We require information from authors about some types of materials, experimental systems and methods used in many studies. Here, indicate whether each material, system or method listed is relevant to your study. If you are not sure if a list item applies to your research, read the appropriate section before selecting a response.

Materials & experimental systems

n/a	Involved in the study	
☒	☐	Antibodies
☒	☐	Eukaryotic cell lines
☒	☐	Palaeontology and archaeology
☒	☐	Animals and other organisms
☒	☐	Human research participants
☒	☐	Clinical data
☒	☐	Dual use research of concern

Methods

n/a	Involved in the study	
☒	☐	ChIP-seq
☒	☐	Flow cytometry
☒	☐	MRI-based neuroimaging
